# Acute Presentation of Large Size Clear Cell Ovarian Carcinoma as Double Torsed Ovarian Tumor

**DOI:** 10.3390/medicina58010089

**Published:** 2022-01-07

**Authors:** Diana Bužinskienė, Vilius Rudaitis, Karolina Misevičiūtė

**Affiliations:** 1 Faculty of Medicine, Vilnius University, LT-03101 Vilnius, Lithuania; diana.buzinskiene@santa.lt (D.B.); vilius.rudaitis@santa.lt (V.R.); 2 Center of Obstetrics and Gynecology, Vilnius University Hospital Santaros Klinikos, LT-08661 Vilnius, Lithuania

**Keywords:** clear cell carcinoma, torsion, ovarian carcinoma, large tumor

## Abstract

We report a 46-year-old patient who presented to the emergency department with complaints of acute abdominal pain, nausea, and vomiting. An abdominal CT scan revealed a large (207 × 155 × 182 mm) thin-walled inhomogeneous tumor connected to the uterus and right ovary. Emergency surgery laparotomy was performed. Inside the abdominal cavity, a 30 × 30 cm heterogenous, dark blue tumor in the right adnexa area, torsed two times, weighing 3700 g was found. Histological examination revealed right ovary clear cell carcinoma. We emphasize the rare nature of the histology and presentation of this case report. Ovarian clear cell carcinomas are relatively rare malignancies, presenting in 5 to 10% of ovarian malignant tumors in the west.

## 1. Introduction

Ovarian cancer is the leading cause of death and is the second most common cancer among gynecological cancers [1]. On the other hand, an ovarian torsion is a frequent gynecological emergency, which affects about 10 out of 100,000 women of reproductive age [2]. Ovarian torsion is difficult to diagnose accurately, thus early surgery is essential to preventing loss of ovarian and tubal function. The current literature supports the idea that the existence of an ovarian mass and its size are the main risk factors of ovarian torsion [3]. Here, we report a 46-year-old woman who presented to the emergency department with signs of acute peritonitis and was diagnosed with a double torsion of a 3700 g ovarian clear cell carcinoma.

## 2. Case Report

A 46-year-old Caucasian woman (parity 0, abortions 0, miscarriages 0) presented to the emergency department with complaints of acute abdominal pain, nausea and vomiting. Previously she underwent two laparoscopic myomectomies. The patient reported irregular menses with long periods of amenorrhea and prolonged menstrual bleeding that lasted for up to one month. Her last period was 20 days ago. No familial cancer history was reported. She had previously been diagnosed with primary arterial hypertension.

On physical examination, the patient was alert, oriented, but felt intense pain. She was examined by the abdominal surgeon, who diagnosed acute peritonitis with the most pain on the left side of the abdomen. There were no urinary or gynecological symptoms. Non-steroid painkillers were prescribed. Laboratory investigation revealed severe anemia: HGB 58 g/L and inflammatory markers: CRB 107 mg/L. Other blood tests were unremarkable.

**Figure 1:**
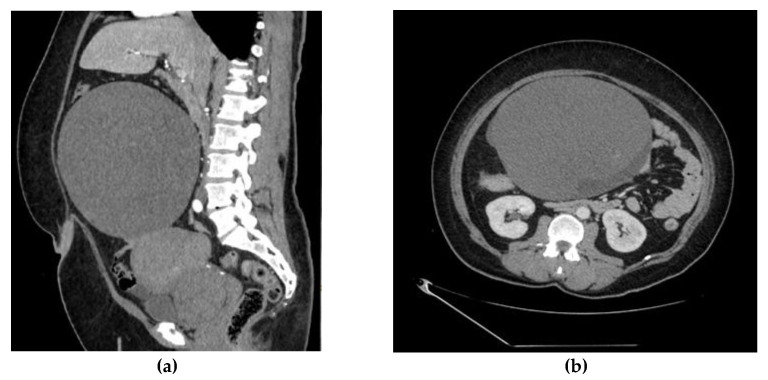
(**a**). Abdominal CT scan shows enlarged and deformed uterus, large (207 × 155 × 182 mm) thin-walled inhomogeneous tumor, connected to uterus and right ovary. (**b**). Abdominal CT scan shows multiple uterine myomas and a large (207 × 155 × 182 mm) thin-walled inhomogeneous tumor.

Abdominal CT scan was performed: an enlarged and deformed uterus with multiple uterine myomas was observed and a large (207 × 155 × 182 mm) thin-walled inhomogeneous tumor connected to the uterus and right ovary was found (Figure 1a,b). the enlarged and deformed uterus with multiple large intramural uterine myomas (about 6–7 cm), which caused prolonged and heavy menstrual bleeding, was the main cause of anemia.

The content of the tumor was similar to hemorrhage. Active extravasation was not present, and a possible right adrenal gland adenoma was detected. A small amount of ascites was also observed.

The patient was redirected to a gynecologist for consultation and treatment. Transvaginal and abdominal ultrasound was performed by the gynecologist, and a 30 × 30 cm tumor with septa, inserts and papillary structures was found in the pelvic cavity. No ascites were detected.

**Figure 2:**
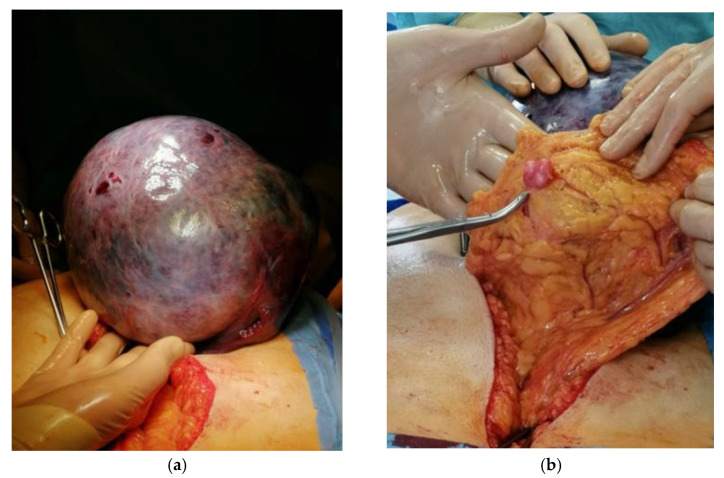
Pictures taken during surgery: (**a**). Right ovary tumor pulled out of the abdominal cavity. (**b**). Parasitic fibroma located on the omentum.

Antibacterial treatment with amoxicillin with clavulanic acid, metronidazole and packed two units of red blood cell transfusion was prescribed to the patient. It was decided to perform an urgent laparotomy. Written informed consent to perform the operation was obtained from the patient. Inside the abdominal cavity, a 30 × 30 cm heterogenous, dark blue tumor in the right adnexa area, torsed two times was found (Figure 2a). Except for parasitic fibromas in the omentum (Figure 2b) there were no other visual or palpable changes found in the abdomen or retroperitoneally. No ascites were found in the abdominal cavity. Total abdominal hysterectomy with right salpingo-oophorectomy was performed.

The tumor was removed without rupture. The content of the tumor did not enter the peritoneal cavity. After removal, it was weighed and was found to be 3700 g. Due to the urgent situation and the late time of the procedure, a frozen section of this tumor was not performed. Multiple, large intramural fibroids were found in the removed uterus on histological examination after the operation.

**Figure 3:**
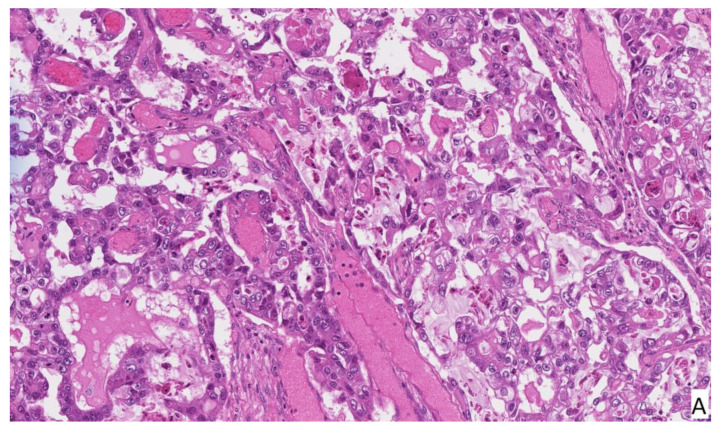

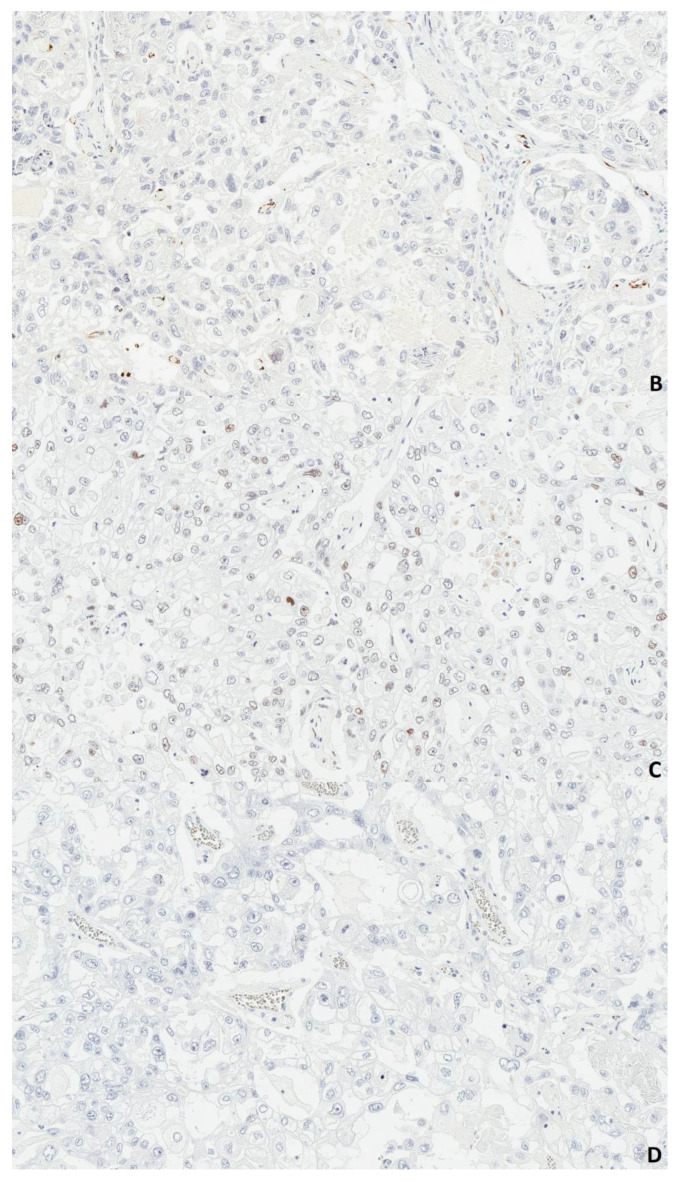

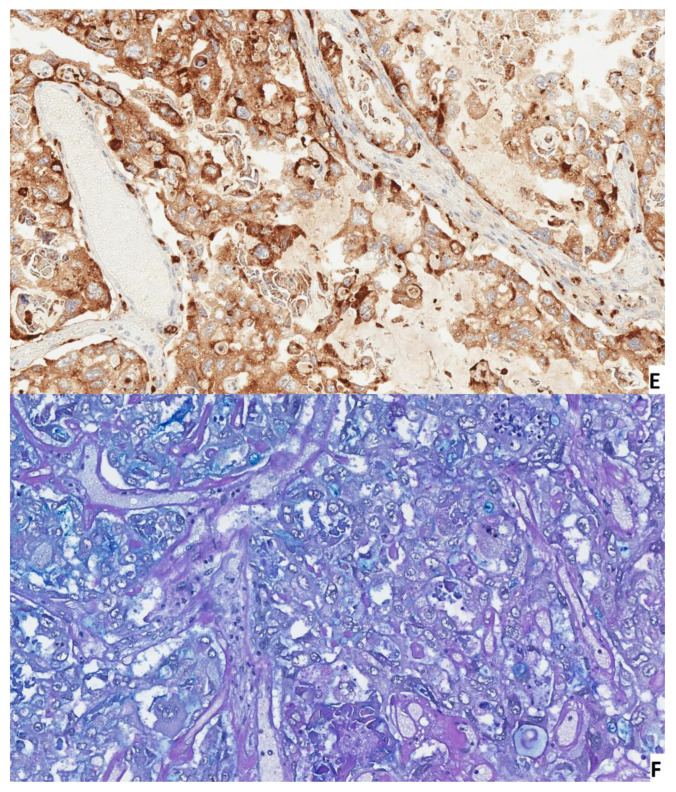
Histopathology of clear cell carcinoma. H&E staining (**A**) shows variation between cells with clear and light eosinophilic cytoplasm. Immunohistochemistry immunoprofile is Wilms tumor 1 (WT-1) negative (**B**), p-53 positive (**C**), HNF1beta negative (**D**), Napsin-A positive (**E**) tumor. Alcian blue/periodic acid–Schiff (AB/PAS) staining (**F**) shows glycogen in clear cells.

The patient left the operating room in stable condition and recovered uneventfully. She was discharged from the hospital after five days of hospitalization without any postoperative complications. Histological examination revealed right ovary clear cell carcinoma TNM stage pT1a with endometrioid gland and stroma formations in the ovary. Clear cell carcinoma phenotype: NapsinA (+++), P53 (+), p16 (++), WT1 (−), HNf-1B (−), SALL4 (−), OCT4 (−) (Figure 3).

The case was presented to the hospital multidisciplinary team. Radiological restaging was recommended. According to the hospital protocol of ovarian cancer staging, a whole body CT was performed. A full body CT scan revealed cystic tumors in the left ovary and a tumor in the left breast that did not accumulate contrast. Small changes were found in the lungs, too small to differentiate. Ultrasound and biopsy of breast tissue were indicated to differentiate tumors. Core needle biopsy of breast tissue revealed fibrocystic changes, left breast Bi RADS 4 category.

After the radiological staging, the patient underwent additional surgery: laparoscopic omentectomy and left salpingoovarectomy with adhesion removal. The surgery and recovery were uneventful. Histological examination revealed follicular cysts in the left ovary and nonspecific changes in the fallopian tube. No changes were found in the omentum, reactive lymphadenopathy was detected. No altered lymph nodes in the abdominal and pelvic cavity were observed in the patient during the radiological examination and surgery. The patient received six cycles of Paclitaxel/Carboplatin chemotherapy.

Currently, the patient is in remission and she is being monitored.

## 3. Discussion

Our patient first presented with acute peritonitis because a typical presentation of ovarian torsion mimics the presentation of acute peritonitis and likewise it is an emergency condition. Up to 15% of patients who were treated surgically for adnexal masses had ovarian torsion [3]. It is also important to note that torsion occurs more commonly with benign tumors. Even though ovarian torsion can occur with ovarian masses of any size, it has been found that masses over five centimeters are of higher risk [3].

Considering gynecological malignancies, ovarian cancer is the leading cause of death and is the second most common gynecological cancer [1]. Ovarian neoplasms have a wide array of subtypes and mostly develop from the surface epithelium and make up about 95% of all ovarian neoplasms [4,5]. Among epithelial ovarian cancers (EOC), the most common subtype is serous tumors (75%), and the common consensus is that they arise from the lesions of the tubal epithelium; however, the exact mechanism of implantation and migration is still unclear [6].

Clear cell carcinoma is characterized by the specific type of glycogen containing clear cell observed during histological examination. Several types of clear cell cancers exist depending on the tissue type in which they developed: renal, uterine and ovarian clear cell carcinomas. Ovarian clear-cell carcinomas (OCCC) are rare malignancies but represent the second most common epithelial ovarian tumors and account for 5–10% of all epithelial ovarian cancers [7]. Since our patient is a Caucasian woman, we found that the incidence differs greatly between different ethnic groups (caucasian 4.8%, black 3.1%, asian 11.1%). Moreover, OCCC incidence has been steadily increasing in Asian countries such as Japan, and is now more than 25% of epithelial ovarian cancers there [8].

OCCC triggers and genesis are unknown. There have been theories that OCCC arises from endometriosis. The association is not clear, but it has been found that patients with ovarian endometrioma or endometriosis have a significantly increased risk of OCCC [9]. This could be the case for our patient as well, since histological examination revealed endometrioid gland and stroma formations in the ovary affected by the tumor. However, endometriosis does not affect disease progression after onset of cancer [10].

Unfortunately, frozen section analysis was not performed on this patient purely because of logistical issues (late time of operation, no on-call pathologist, and poor patient condition). However, the current literature suggests that frozen section analysis is a lot less accurate than the traditional paraffin section examination and might be more beneficial in the diagnosis of borderline tumors. During the initial first operation, the surgeon may choose to perform comprehensive staging to avoid the need for additional surgery in case the final diagnosis turns out to be cancer [11].

Frozen section testing of ovarian masses can be used intraoperatively for the investigation of women with ovarian masses suspected to be early-stage malignancies (11). In practice, the use of frozen section depends on a number of factors, such as the prevalence of cancer within a referred population, the expertise of the gynecologist to perform a surgical staging procedure should the frozen section result prove to be cancer and the ability of the pathologist to interpret frozen sections and for histopathology departments to provide the frozen section service (11).

Adjuvant chemotherapy has been a staple in the treatment of OCCC because of poor later stage prognosis [7]. However, a recent meta-analysis has found that adjuvant chemotherapy does not improve oncologic outcomes of OCCC IA and IB stage, whereas administering adjuvant chemotherapy for IC stage improves 5 year survival [12].

During a multidisciplinary patient meeting, this was discussed, and it was decided that according to radiologic and histologic staging after the first surgery, surgery staging during the laparoscopic surgery was not indicated, thus we did not perform bilateral pelvic lymphadenectomy, para-aortic lymphadenectomy, washing cytology and peritoneal biopsy.

In the case we have described, lymphadenectomy did not affect the patient‘s treatment options or survival. This case represents a real clinical situation and the safe options of how it was solved in our clinic.

According to the scientific literature, pelvic and para-aortic lymphadenectomy in patients with early-stage ovarian clear cell carcinoma (OCCC) was not associated with improved disease-free and overall survival. Patients with pTI–IIb CCC who underwent lymphadenectomy did not show a significant improvement in survival. There was no significant difference in the overall and disease-free survival rates in pTI–IIb CCC patients regardless of the completion of surgical staging lymphadenectomy [13].

Lymphadenectomy improved the survival in those with non-clear cell epithelial ovarian cancer (85.9% to 93.3%, *p* < 0.001), but not in those with clear cell carcinoma, germ cell tumors, sex cord stromal tumors, and sarcomas. Clear cell ovarian cancers (83.8% compared with 86.6%; *p* = 0.31) did not show a statistically significant benefit toward better survival associated with lymphadenectomy [14].

In our presented case, we thought that ours was the most rational approach concerning our patient’s treatment, despite the fact that staging was not complete.

A combination of debulking surgery and platinum based chemotherapy is the current standard of OCCC treatment [15]. Despite a good initial response to platinum based chemotherapy, a lot of patients experience relapse and develop resistance to chemotherapy [16]. Quite recently there has been a prospective phase-II trial, which reported favorable results for adjuvant whole abdominal radiotherapy using intensity-modulated radiotherapy in FIGO stage III ovarian cancer [15]. Yet, there is still not enough efficacy data to support the use of radiotherapy in such cases.

## 4. Conclusions

Ovarian clear cell carcinomas are relatively rare malignancies; however, the incidence rate has been steadily increasing in Asian countries over the last decade. The exact mechanism of ovarian clear cell carcinoma development is still unclear; however, the recent literature has found compelling evidence that shows endometriosis as a key factor in tumor genesis. A lot of patients in cases of advanced OCCC develop resistance to usual chemotherapy and have recurrent disease, which reduces overall survival. Adjuvant chemotherapy in early stages (IA and IB) of ovarian clear cell carcinoma has been found to be ineffective in increasing overall survival; however, adjuvant chemotherapy increased 5-year survival in the IC stage.

## References

[1] Siegel R., Naishadham D., Jemal A. (2012). Cancer statistics, 2012. CA Cancer J. Clin..

[2] Yuk J.-S., Kim L.Y., Shin J.-Y., Choi D.Y., Kim T.Y., Lee J.H. (2015). A national population-based study of the incidence of adnexal torsion in the Republic of Korea. Int. J. Gynecol. Obstet..

[3] Huang C., Hong M.-K., Ding D.-C. (2017). A review of ovary torsion. Tzu-chi Med. J..

[4] Desai A., Xu J., Aysola K., Qin Y., Okoli C., Hariprasad R., Chinemerem U., Gates C., Reddy A., Danner O. (2014). Epithelial ovarian cancer: An overview. World J. Transl. Med..

[5] Nik N.N., Vang R., Shih I.-M., Kurman R.J. (2014). Origin and pathogenesis of pelvic (ovarian, tubal, and primary peritoneal) serous carcinoma. Annu. Rev. Pathol..

[6] Oseledchyk A., Leitao M.M., Konner J., O’Cearbhaill R.E., Zamarin D., Sonoda Y., Gardner G.J., Roche K.L., Aghajanian C.A., Grisham R.N. (2017). Adjuvant chemotherapy in patients with stage I endometrioid or clear cell ovarian cancer in the platinum era: A Surveillance, Epidemiology, and End Results Cohort Study, 2000–2013. Ann. Oncol..

[7] Fujiwara K., Shintani D., Nishikawa T. (2016). Clear-cell carcinoma of the ovary. Ann. Oncol..

[8] Pearce C.L., Templeman C., Rossing M.A., Lee A., Near A.M., Webb P.M., Nagle C.M., Doherty J.A., Cushing-Haugen K.L., Wicklund K.G. (2012). Association between endometriosis and risk of histological subtypes of ovarian cancer: A pooled analysis of case–control studies. Lancet Oncol..

[9] Kim H.S., Kim M.A., Lee M., Suh D.H., Kim K., No J.H., Chung H.H., Kim Y.B., Song Y.S. (2015). Effect of Endometriosis on the Prognosis of Ovarian Clear Cell Carcinoma: A Two-Center Cohort Study and Meta-analysis. Ann. Surg. Oncol..

[10] Bogani G., Ditto A., Lopez S., Bertolina F., Murgia F., Pinelli C., Chiappa V., Raspagliesi F. (2020). Adjuvant chemotherapy vs. observation in stage I clear cell ovarian carcinoma: A systematic review and meta-analysis. Gynecol. Oncol..

[11] Ratnavelu N.D., Brown A.P., Mallett S., Scholten R.J., Patel A., Founta C., Galaal K., Cross P., Naik R. (2016). Intraoperative frozen section analysis for the diagnosis of early stage ovarian cancer in suspicious pelvic masses. Cochrane Database Syst. Rev..

[12] Morrison J., Haldar K., Kehoe S., Lawrie T.A. (2012). Chemotherapy versus surgery for initial treatment in advanced ovarian epithelial cancer. Cochrane Database Syst. Rev..

[13] Suzuki S., Kajiyama H., Shibata K., Ino K., Nawa A., Sakakibara K., Matsuzawa K., Takeda A., Kinoshita Y., Kawai M. (2008). Is there any association between retroperitoneal lymphadenectomy and survival benefit in ovarian clear cell carcinoma patients?. Ann. Oncol..

[14] Chan J.K., Munro E.G., Cheung M.K., Husain A., Teng N.N., Berek J.S., Osann K. (2007). Association of Lymphadenectomy and Survival in Stage I Ovarian Cancer Patients. Obstet. Gynecol..

[15] Wiggans A.J., Cass G.K., Bryant A., Lawrie T.A., Morrison J. (2015). Poly(ADP-ribose) polymerase (PARP) inhibitors for the treatment of ovarian cancer. Cochrane Database Syst. Rev..

[16] Arians N., Kieser M., Benner L., Rochet N., Katayama S., Sterzing F., Herfarth K., Schubert K., Schröder L., Leitzen C. (2017). Adjuvant Intensity Modulated Whole-Abdominal Radiation Therapy for High-Risk Patients with Ovarian Cancer (International Federation of Gynecology and Obstetrics Stage III): First Results of a Prospective Phase 2 Study. Int. J. Radiat. Oncol. Biol. Phys..

